# Exploring the leishmanicidal potential of terpenoids: a comprehensive review on mechanisms of cell death

**DOI:** 10.3389/fcimb.2023.1260448

**Published:** 2023-09-19

**Authors:** Ana Carolina Jacob Rodrigues, Amanda Cristina Machado Carloto, Manoela Daiele Gonçalves, Virgínia Márcia Concato, Mariana Barbosa Detoni, Yasmin Munhoz dos Santos, Ellen Mayara Souza Cruz, Maria Beatriz Madureira, Angélica Paulina Nunes, Maria Fernanda Maya Kuriki Pires, Natália Concimo Santos, Rafaela Evangelista dos Santos Marques, Danielle Lazarin Bidoia, Fabiano Borges Figueiredo, Wander Rogério Pavanelli

**Affiliations:** ^1^ Laboratory of Immunoparasitology of Neglected Diseases and Cancer, Department of Pathological Sciences, State University of Londrina, Londrina, Brazil; ^2^ Cell Biology Laboratory, Carlos Chagas Institute- Fiocruz, Curitiba, Brazil; ^3^ Chemistry Department, Exact Sciences Center, State University of Londrina, Londrina, Brazil; ^4^ Laboratory of Experimental Immunoparasitology, Department of Pathological Sciences, State University of Londrina, Londrina, Brazil; ^5^ Laboratory for Metabolic Disorders of Reproduction, Department of Pathological Sciences, State University of Londrina, Londrina, Brazil

**Keywords:** *Leishmania*, terpenes, treatment, oxidative stress, immunomodulation

## Abstract

Leishmaniasis is a neglected tropical disease with a wide spectrum of clinical manifestations, ranging from visceral to cutaneous, with millions of new cases and thousands of deaths reported each year. The species of *Leishmania* and the immune response of the host determine the severity of the disease. Leishmaniasis remains challenging to diagnose and treat, and there is no vaccine available. Several studies have been conducted on the use of herbal medicines for the treatment of leishmaniasis. Natural products can provide an inexhaustible source of chemical diversity with therapeutic potential. Terpenes are a class of natural products derived from a single isoprene unit, a five-carbon compound that forms the basic structure of isoprenoids. This review focuses on the most important and recent advances in the treatment of parasites of the genus *Leishmania* with different subclasses of terpenes. Several mechanisms have been proposed in the literature, including increased oxidative stress, immunomodulatory role, and induction of different types of parasite cell death. However, this information needs to be brought together to provide an overview of how these compounds can be used as therapeutic tools for drug development and as a successful adjuvant strategy against *Leishmania* sp.

## 1 Background

Natural products are secondary metabolites found in various parts of plants, including roots, fruits, leaves, stems, and seeds. They represent a valuable resource for the discovery of new bioactive molecules with potential pharmacological effects and for the development of new drugs through modifications of the molecular structure (Gervazoni et al., 2020). Natural products can be classified into three main groups: phenolic compounds, nitrogen-containing compounds, and terpenes (Jamwal et al., 2018). Terpenes, derived from isoprene (2-methyl-1,3-butadiene), a hemiterpene with 5 carbon atoms, have antimicrobial, anti-inflammatory, antitumor, and antioxidant properties (Do Nascimento et al., 2020). These compounds are found in the leaves and flowers of plants such as *Origanum vulgare*, which produce essential oils (EO) containing components such as thymol and carvacrol. Terpenes are also known for their potential therapeutic effects against various diseases, including parasitic infections such as leishmaniasis (Strothmann et al., 2022; Tomiotto-Pellissier et al., 2022; Alves et al., 2023).

Neglected tropical diseases (NTDs) encompass a range of infections caused by various types of infectious and parasitic agents, including protozoa, viruses, bacteria, and helminths. These diseases impact over one billion people worldwide, particularly impoverished populations in parts of Africa, Asia, and Latin America. This presents a significant public health concern (Luna et al., 2019). Despite this, these diseases receive limited attention from major pharmaceutical companies, leading to insufficient investment in the advancement of new treatments. Of the NTDs, leishmaniasis, identified as predominantly zoonotic, includes a group of diseases caused by protozoa of the genus *Leishmania* transmitted between mammalian hosts by female phlebotomines. This transmission occurs *via* insects of the genus *Lutzomyia* in the Americas or *Phlebotomus* in the Old World. The *Leishmania* genus comprises about 20 species, and the disease can be classified as cutaneous/tegumentary or visceral, based on the species involved. The cutaneous form involves skin and mucous membranes, and often results in disfiguring lesions, while the visceral form affects internal organs and can lead to death if untreated (Diaz-Albiter et al., 2018; Mendoza-Roldan et al., 2022).

*Leishmania* species exhibit varying degrees of virulence, and different strains of the same species may differ in infectivity, virulence, and metastasis phenotypes. Cutaneous leishmaniasis (CL) is caused by dermotropic species, including *L. tropica*, *L. major*, and *L. mexicana*, which can cause less severe lesions that are self-healing in a short time. However, *Viannia* complex (*L. braziliensis*, *L. guyanensis*, and *L. panamensis*) are associated with ulcerative lesions that are generally more prevalent in immunocompromised individuals (Burza et al., 2018; Ait Maatallah et al., 2022). The diffuse manifestation of the cutaneous form is rarer and involves species such as *L. amazonensis*, *L. aethiopica*, and *L. mexicana*. This condition reflects the absence of a cellular immune response by the host. Visceral leishmaniasis (VL) is caused exclusively by viscerotropic species such as *L. donovani* and *L. infantum*. In cases of treatment failure with *L. donovani*, this species can cause post-kalavar dermal leishmaniasis, a dermal complication of VL, suggesting a loss of its viscerotropic nature (Ait Maatallah et al., 2022).

In general, the cell cycle of the disease begins after blood meal infection of the female sandflies. During this process, the vector’s saliva contains endonucleases that can digest neutrophil extracellular networks (NETs) and inhibit blood clotting, allowing the promastigotes to survive the microenvironment (Chagas et al., 2014; Bates, 2018). These promastigotes are then phagocytosed by macrophages and other cells of the monocytic phagocytic system to form amastigotes (tissue stage). Inside the macrophages, the amastigotes, protected from the host’s immune response and exposed to microbicidal factors such as the hydrolytic enzymes of the phagolysosome, undergo cell replication. After enhanced replication, the parasite can break through the macrophages and is phagocytosed by new cells of the monocytic system (Conceição-Silva and Morgado, 2019), thus establishing a parasitic infection.

This review focused on studying the effectiveness of various terpenoid classes and the cellular mechanisms of cell death they involve in the context of treating cutaneous and visceral leishmaniasis. We looked through the digital databases of PubMed and ScienceDirect for the updated research published between 2018 and 2022. The terms or keywords used in the search were “terpene/*leishmania*,” “monoterpene/*leishmania*,” sesquiterpene/*leishmania*,”. “diterpene/*leishmania*,” and “triterpene/*leishmania*,”, however, the latter did not present results that fit this work. The chosen articles were closely scrutinized. We removed studies that presented data of low quality, erroneous, or not considered relevant to the goal of the review, as well as those reporting anti-leishmanial activity of extracts, after individually reading the titles and abstracts of all papers retrieved by the search. The 43 studies that were ultimately chosen and downloaded are shown in this study.

### 1.1 Current chemotherapy targets

NTDs transmitted by vectors, along with their associated co-infections, present notable challenges in treatment due to various constraints. While leishmaniasis can be successfully treated if detected early and managed appropriately, the effectiveness of treatment is influenced by factors like specific parasite species, host characteristics, and geographical location (Madusanka et al., 2022). Furthermore, these challenges encompass issues like toxicity, poor absorption, drug resistance, insufficient efficacy, and prolonged treatment duration (Nweze et al., 2021). Various therapeutic approaches are available to combat leishmaniasis, including chemotherapy, cryotherapy, thermotherapy, and alternative methods that are less commonly used. Presently, the chemotherapeutic treatment of leishmaniasis relies on primary medications like sodium stibogluconate (Pentostam) and meglumine antimoniate (Glucantime), alongside secondary options such as pentamidine isethionate (Pentamidine), amphotericin B (AmB) in forms like Fungizone or Ambisome, miltefosine, paromomycin sulfate (Aminosidine), and azole drugs (Madusanka et al., 2022).

The antimonial medication Glucantime was formulated over 70 years ago and continues to be employed for addressing both visceral and tegumentary leishmaniasis, with varying dosages tailored to the clinical manifestation. This medication can activate the apoptosis mechanism within the parasite by impeding the synthesis of crucial enzymes like trypanothione reductase and phosphatidylcholine. These enzymes play a pivotal role in upholding the parasite’s plasma membrane integrity (Barroso et al., 2021; Santiago et al., 2021). AmB, on the other hand, exhibits a strong attraction to the steroidal components of the parasite’s plasma membrane, particularly ergosterol. Once the interaction between the drug and the membrane takes place, it disrupts the membrane’s integrity, resulting in increased permeability, ionic imbalances, and consequential alterations that ultimately disorganize the parasite’s plasma membrane. This disruption ultimately leads to the death of the parasite (Santiago et al., 2021). Beyond pharmacological approaches, managing the protozoan infection also involves non-pharmacological methods such as rest and a nutritious diet.

Miltefosine serves as an additional medication for treating the disease, particularly its visceral form. The drug effectively functions by inhibiting the synthesis of phospholipids and sterols within the parasite cell membrane, inducing apoptosis in both *Leishmania* promastigotes and amastigotes (Palić et al., 2022). However, exceptions exist in specific scenarios, such as pregnant women, individuals with renal insufficiency, children under the age of one, and those with other underlying conditions. For such cases, the recommendation leans toward AmB or liposomal AmB (Silva et al., 2013).

The World Health Organization recently aligned with the Sustainable Development Goals to accelerate the control and elimination of NTDs by 2030. In addressing leishmaniasis, their primary focus revolves around enhancing treatments, diagnostics, and vaccines against the *Leishmania* parasite (WHO, 2021). Given the prevailing challenges, the demand for more accessible and efficient treatments in the current market is of utmost importance. Consequently, the exploration of novel therapeutic options like terpenes holds significant potential for advancing improved strategies in managing this disease (Passero et al., 2018).

## 2 Terpenic compounds with anti-*leishmania* activity

Terpenes are a class of natural compounds derived from a single molecule of isoprene. With over 55,000 different structurally diverse compounds, they are responsible for imparting properties such as flavor, aroma, and pigment to plants (Ninkuu et al., 2021). Components of primary and secondary plant metabolism, terpenes play essential roles in intra- and intercellular processes including photosynthesis and respiratory chains. Additionally, they act as thermal protectants, signaling agents, and provide protection against pests and insects (Cox-Georgian et al., 2019). Terpenes are also the primary constituents of essential oils, which have extensive applications in the pharmaceutical industry (Sommano et al., 2020).

Chemically, terpenes can be defined as natural alkenes. They contain a carbon-carbon double bond, classifying them as unsaturated hydrocarbons (Ninkuu et al., 2021). When a terpene contains oxygen, it is called a terpenoid. Terpenoids can have various chemical functions, including acids, alcohols, aldehydes, ketones, ethers, phenols, or terpenic epoxides (Perveen, 2018). Despite their structural differences, all terpenes/terpenoids are basically composed of five carbons linked in a head-tail arrangement (bonds 1-4), defining the isoprene rule. However, there are variations in the linking of isoprenes, which can be head-head (1-1) or tail-tail (4-4) (Hillier and Lathe, 2019).

Terpenes are synthesized by the enzyme terpene synthase (TPS) and undergo modification such as hydroxylation, acetylation, glycosylation, or dehydrogenation, resulting in chemically diverse terpenoid compounds (Rudolf and Chang, 2020). The synthesis of terpenes occurs through the biochemical utilization of isopentenyl diphosphate (IPP) or its isomer dimethylallyl diphosphate (DMAPP), from which the carbons of terpenes originate. These precursors generate various terpenes through two distinct metabolic pathways: the mevalonate pathway (MVA), which occurs in the cytosol, and the 1-deoxylulose 5-phosphate pathway (DXP) (Chatzivasileiou et al., 2019; Tetali, 2019).

Terpenoids are classified according to the number and structural organization of the carbons. This results from the linear arrangement of the isoprene units, followed by cyclization and reorganization of the carbon skeleton. Consequently, the nomenclature is determined by the number of carbon atoms. Hemiterpenes, for instance, consist of terpenes with a five-carbon (C5) isoprene unit in their structure. Monoterpenes are molecules composed of ten carbon atoms (C10) and are predominantly volatile compounds due to their lower molecular mass. Larger molecules are named based on the number of carbons present: sesquiterpenes (C15), diterpenes (C20), triterpenes (C30), tetraterpenes or carotenoids (C40), and polyterpenes (C>40) (Tetali, 2019). Additionally, terpenes can be further classified based on the degree of cyclization of the molecule as acyclic (open molecules), monocyclic, or bicyclic (Mosquera et al., 2021).

### 2.1 Monoterpenes display leishmanicidal effects through the disruption of parasite metabolism

**Figure 1:**
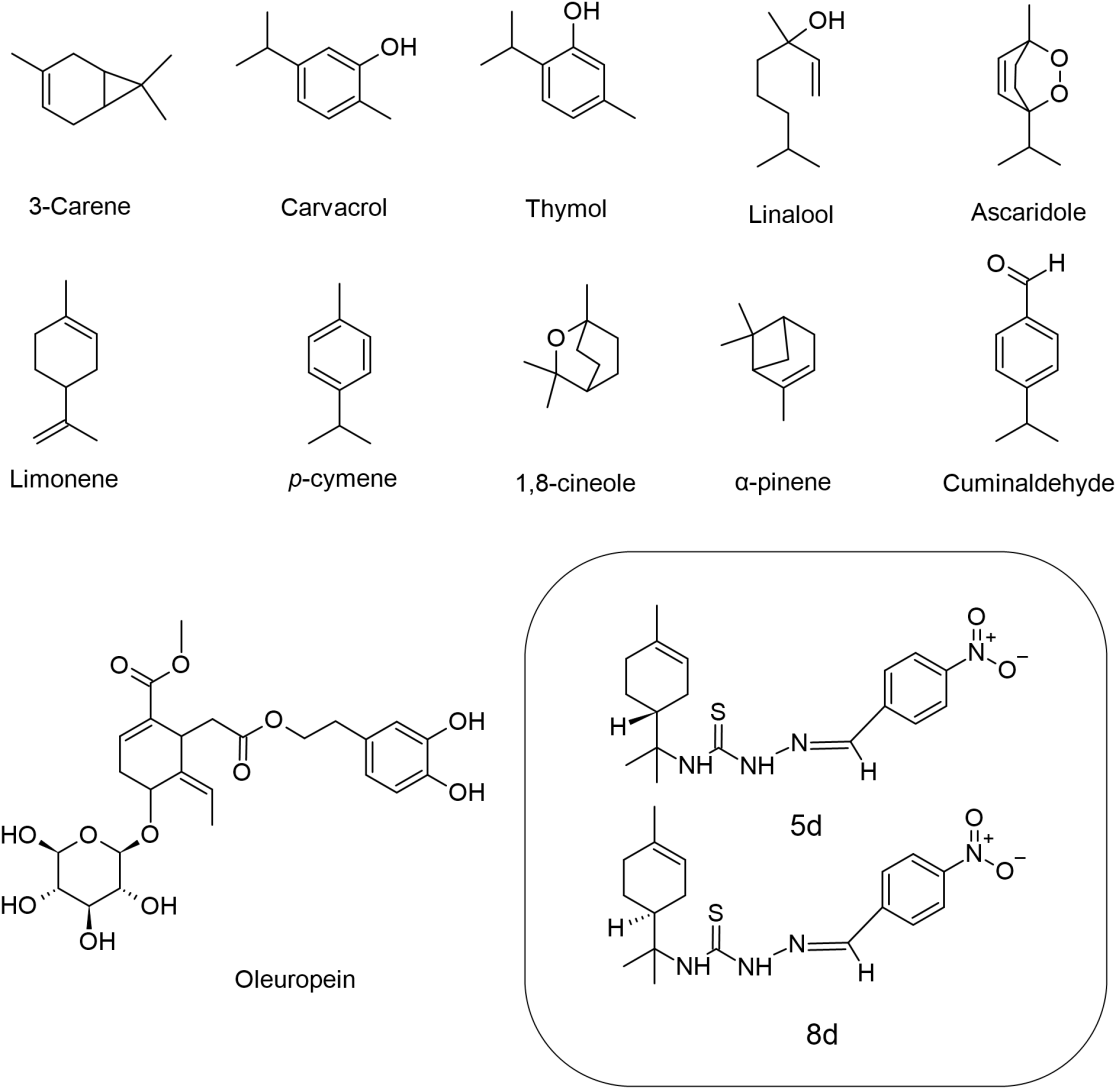
Chemical structures of the monoterpenes that showed activity against *Leishmania* spp.

**Figure 2:**
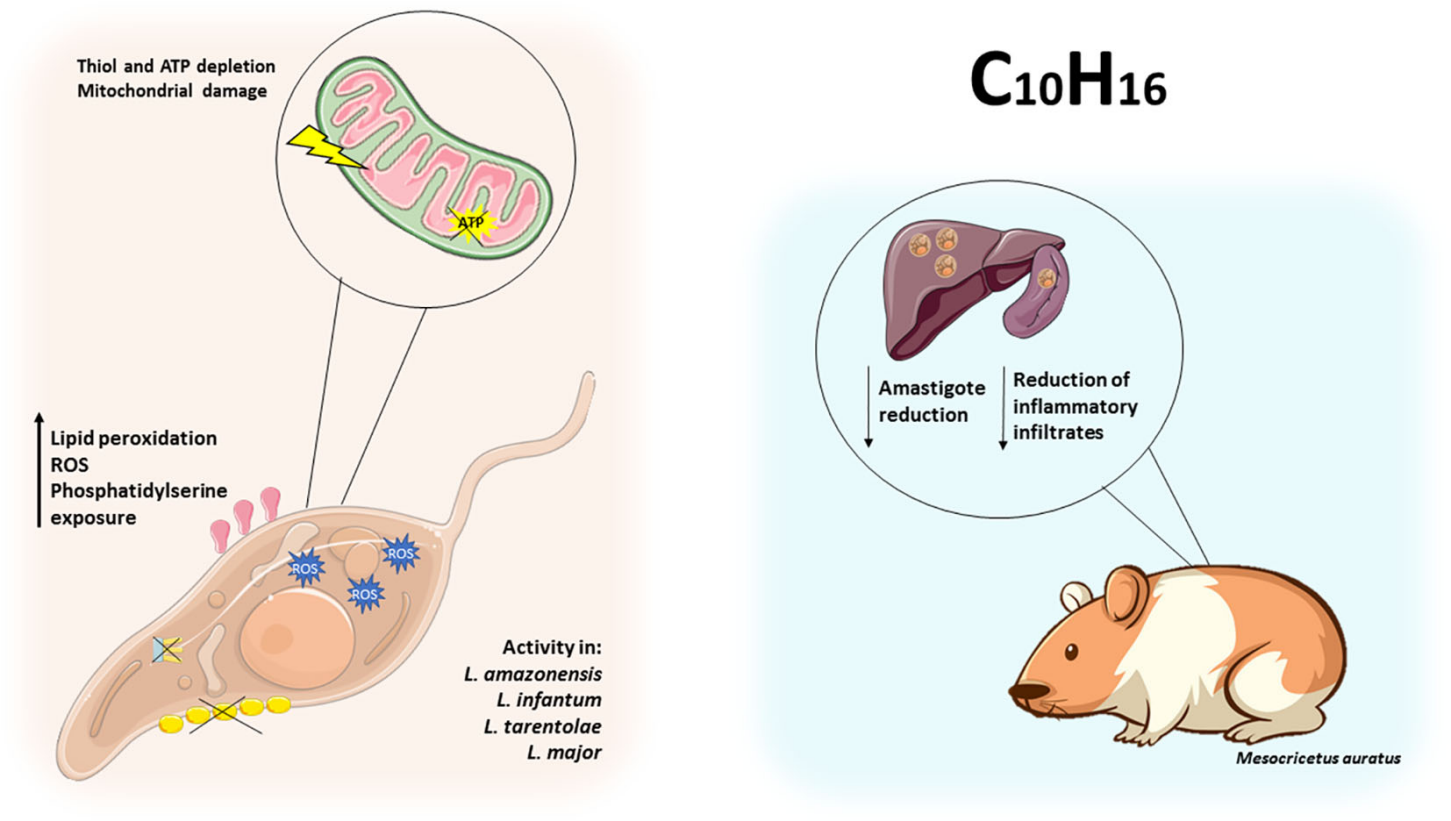
Monoterpenes exert leishmanicidal activity by disrupting parasite metabolism. Monoterpenes alter the metabolism of *Leishmania* spp. by increasing lipid peroxidation, ROS, thiol and ATP depletion, leading to mitochondrial damage and phosphatidylserine exposure in promastigotes. In hamsters (*Mesocricetus auratus*), thymol reduced the number of amastigotes in the liver and kidneys, and carvacrol reduced the local inflammatory response.

Monoterpenes (C_10_H_16_) are composed only of carbon and hydrogen atoms, and represent a great number of organic compounds containing two isoprene units (**Figure 1**). Monoterpenes are ubiquitous natural products that are abundant in plants and primarily contribute to the flavor and aroma properties commonly observed in their constituents such as flowers and fruit (Kabir et al., 2020). The biological activity of these compounds against *Leishmania* sp. (**Table 1** and **Figure 2**) will be discussed in this section.

**Table 1:** Summary of the biological activity of monoterpenes.

Monoterpene	*Leishmania* spp.	IC_50_ promastigotes	IC_50_ amastigotes	References
3-carene (micellar systems)	*L. braziliensis* (Tegumentary and mucosal leishmaniasis)	45.66µg/mL	–	(Silva et al., 2023)
Carvacrol free	*L. infantum* (Visceral leishmaniasis) *L. tarentolae* (Non-pathogenic) *L. major* (Cutaneous leishmaniasis)	9.80 µg/mL 11.60 µM 5.80 µg/mL	- - -	(Youssefi et al., 2019) (Monzote et al., 2018) (Carvalho et al., 2021)
Carvacrol nanoencapsulated (hydrogel+poloxamer)	*L. amazonensis* (Cutaneous leishmaniasis)	18.68 µg/mL	35.08 µg/mL	(Costa et al., 2023)
Carvacrol nanoencapsulated (nanostructured lipids)	*L. amazonensis* (Cutaneous leishmaniasis)	28.20 µg/mL (48h) 19.65 µg/mL (72h)	–	(Galvão et al., 2020)
Ascaridole	*L. tarentolae* (Non-pathogenic) *L. donovani* (Visceral leishmaniasis)	24.50 µg/mL 2.47 µM	- 2.00 µM	(Monzote et al., 2018) (Sarkar et al., 2022)
Limonene	*L. major* (Cutaneous leishmaniasis)	16.00 µg/mL	–	(Carvalho et al., 2021)
Limonene+carvacrol	*L. major* (Cutaneous leishmaniasis)	15.40 µg/mL	–	(Carvalho et al., 2021)
4-nitrobenzaldehyde thiosemicarbazones (limonene)	*L. amazonensis* (Cutaneous leishmaniasis)	2.4-8.7 µM	–	(Almeida Batista et al., 2019)
1,8-cineole	*L. amazonensis* (Cutaneous leishmaniasis)	–	–	(Santana et al., 2020)
*p*-cymene	*L. amazonensis* (Cutaneous leishmaniasis)	–	–	(Santana et al., 2020)
⍺-pinene	*L. amazonensis* (Cutaneous leishmaniasis)	–	–	(Santana et al., 2020)
Cuminaldehyde	*L. major* (Cutaneous leishmaniasis)	19.70 µg/mL	9.30 µg/mL	(Mohamadi et al., 2023)
Oleuropein	*L. major* (Cutaneous leishmaniasis)	50 µg/mL	–	(Elamin and Al-Maliki, 2014)

In column 1: type of monoterpene, column 2: species of Leishmania on which the compound showed activity, column 3: IC_50_ of promastigotes, column 4: IC_50_ of amastigotes and column 5: references.

3-Carene (3CR) is a bicyclic monoterpene present in many types of plants with demonstrated leishmanicidal activity in the literature. The aim of this study was to prepare micellar systems in an attempt to optimize the delivery of the compound with lower toxicity and higher bioavailability. The authors developed a monomeric micellar system using a hydrophilic poloxamer (P407) combined with a hydrophobic L81 to form smaller and stable micelles with the monoterpene 3CR. Several micellar groups were tested and the one with the lowest concentration of poloxamers (5/0.3%) was selected due to its better physicochemical parameters and significant anti-promastigote activity, with a half-maximal concentration inhibition (IC_50_) value of 45.66 μg/mL in *Leishmania braziliensis*. Based on the selectivity index (SI) in micellar systems, 3CR was three times more toxic to the parasite than to the J774.1 cell line (murine macrophages). However, L81 appears to exhibit some degree of cellular toxicity, and the authors emphasize the need for further testing of this nanocarrier (Silva et al., 2023).

Similarly, thermosensitive hydrogels of the triblock copolymers of poloxamer 407 (P407) and 188 (P188) were combined with the monoterpene carvacrol (HG407/188CA). HG407/188CA and free carvacrol were tested on *Leishmania amazonensis*. HG407/188CA showed IC_50_ values similar to free carvacrol of 18.68 µg/mL in promastigote forms and 35.08 µg/mL in intracellular amastigotes. The SI against *L. amazonensis* amastigotes, however, was 8-fold higher than against murine peritoneal macrophages and L929 (murine fibroblasts) compared to carvacrol alone. This suggests that the hydrogel reduced the cytotoxicity of carvacrol in these cells. The authors suggest that nanocarrier systems allow the use of compounds at lower doses and with greater efficacy, especially for monoterpenes that have greater volatility and low solubility in water, facilitating the formulation of pharmaceutical products against leishmaniasis (Costa et al., 2023). Another type of carrier is nanostructured lipids (NLCs), which have been associated with carvacrol against *L. amazonensis*. When encapsulated, carvacrol showed an IC_50_ of 28.2 µg/mL at 48 h and 19.65 µg/mL at 72 h, in addition to lower toxicity in THP-1 cells (human monocytic leukemia cell line) with an SI of 3.74 compared to the free compound (SI 2.3 at 72 h). In an *in vivo* model, carvacrol shows enterohepatic circulation, a long half-life and low renal clearance, characteristics shared by many existing drugs, which may allow reabsorption of part of the treatment in the gut. When nano-encapsulated, the pharmacokinetics of carvacrol were very similar except for the residence time, which was longer. This may favor the distribution of the compound in the body. According to the authors, the active compound may be protected from metabolism and excretion when encapsulated by excipients (Galvão et al., 2020).

Youssefi et al. (2019) demonstrated the activity of carvacrol, thymol, and linalool, against *L. infantum*. Only thymol and carvacrol were found to have significant *in vitro* activity against promastigote forms of the parasite, with IC_50_ values of 7.2 μg/mL and 9.8 μg/mL, respectively. The highest concentration of thymol used (10 μg/mL) was comparable to that of glucantime. In hamsters (*Mesocricetus auratus*) infected with *L. infantum*, treatment with thymol at a dose of 100 mg/kg was able to reduce the number of amastigotes in the liver of the animals. However, it did not suppress the inflammation caused by the disease. On the other hand, when the animals were treated with carvacrol, there was no reduction in the parasite load in the liver or spleen. However, there was a reduction in inflammatory infiltrates similar to that observed with glucantime. These inflammatory infiltrates are largely responsible for affecting the portal tracts of the liver and accumulating within the organ (Youssefi et al., 2019).

Monzote et al. (2018) evaluated the activity of three major components of *Chenopodium ambrosioides* L. EO, carvacrol, caryophyllene oxide (Caryo), a sesquiterpene, and ascaridole (Asc), against *L. tarentolae*, a parasite that is non-pathogenic to humans but causes disease in other animal species. The experiments yielded IC_50_ values of 24.5 μM, 11.6 μM and 36.0 μM for Asc, carvacrol and Caryo, respectively. Asc induced an increase in superoxide radical production resulting in mitochondrial uncoupling after prolonged incubation with the treatment. All treatments led to thiol depletion after prolonged incubation with the parasite. The results suggest that the compounds act on the mitochondrial pathways of *Leishmania* spp. in a time-dependent manner (Monzote et al., 2018). In another study, Asc showed potent leishmanicidal activity against promastigote and amastigote forms of *L. donovani*, with IC_50_ values of 2.47 µM and 2.00 µM, respectively. It disrupted the redox balance of the parasite by increasing the generation of reactive oxygen species (ROS), lipid peroxidation, and thiol depletion. This monoterpene also inhibited the glycolytic pathway in promastigotes, resulting in adenosine triphosphate (ATP) depletion. The cellular stress induced by ascaridole treatment resulted in increased labeling of annexin V, a marker that binds to phosphatidylserine when exposed on the cell surface, and caused cell cycle arrest of promastigotes in the G0/G1 phase (Sarkar et al., 2022).

The leishmanicidal activity of two well-known monoterpenes, limonene and carvacrol, was tested. Both compounds showed direct activity against *L. major* with IC_50_ values of 16.00 and 5.80 μg/mL, respectively. The authors also investigated the activity of the combination limonene e carvacrol (Lim-Car), where the 4:1 Lim-Car fraction (IC_50_ 15.40 μg/mL) showed promising results against infected macrophages and an SI of 22.4 for the parasite. The combination treatment also induced macrophage activity and molecular docking predictions showed that the Lim-Car compounds act directly on the parasite. The Lim-Car combination thus represents a better alternative, being less toxic and more effective than the monoterpenes tested alone (Carvalho et al., 2021). In another study, the authors tested whether the leishmanicidal activity of limonene was indeed due to the monoterpene moiety of the compound. Therefore, analogues derived from R-(+)- and S-(-)-limonene were evaluated against *L. amazonensis*. All investigated series showed anti-leishmanial activity; however, the derived 4-nitrobenzaldehyde thiosemicarbazones showed the highest activity with IC_50_ values ranging from 2.4 to 8.7 µM, with the S-(-) derivative showing high selectivity. The results suggest that the monoterpene moiety in the limonene analogs plays an important role in the activity of the molecule, and changes in conformation and stereochemistry can alter the biological activity of the compounds (Almeida Batista et al., 2019).

The oleoresin of *Protium* sp. is used in traditional medicine due to its high content of mono- and triterpenes. In this context, Santana et al. (2020) evaluated the leishmanicidal activity of *Protium altsonii* (PaEO) and *P. hebetatum* (PhEO) oleoresins and three of their major volatile components, 1,8-cineole, *p*-cymene and α-pinene. Initially, the authors found that PaEO and PhEO layers reduced the viability of promastigote forms of *L. amazonensis*. As for the isolated compounds, only *p*-cymene reduced parasite viability at the highest concentrations. In addition, treatment with 1,8-cineole reduced the levels of triglycerides, sterols and diacylglycerols, suggesting that this compound may interfere with the lipid metabolism of the parasite, causing it to use up energy reserves under conditions of metabolic stress. They also found that all three monoterpenes showed dose-dependent activity against the amastigote form of the parasite. This effect was attributed to the likely need of the cell to metabolize these compounds to produce a derivative toxic to the parasite. Furthermore, none of the extracts and isolated compounds were toxic to murine macrophages (Santana et al., 2020).

Cuminaldehyde is an oxidized monoterpenoid aldehyde and the main component of cumin seeds (*Bunium persicum*). It has shown promising biological effects. Molecular docking analysis indicated that cuminaldehyde binds to interleukin 12p40 (IL-12p40) and tumor necrosis factor alpha (TNF-α). Consistent with the prediction, quantitative polymerase chain reaction (qPCR) data showed that treatment with cuminaldehyde (IC_50_ of 19.7 and 9.3 µg/mL) against *L. major* promastigotes and amastigotes negatively regulates the Th2 phenotype. When cytokines were measured, a significant increase in Th1 pattern cytokines (IFN-γ, IL-12p40, TNF-α) was observed, suggesting that this molecule is a potent immunomodulator in leishmaniasis (Mohamadi et al., 2023). Oleuropein, a glycosylated seco-iridoid, is a natural compound found in olive leaves (*Olea europaea*). In a study, oleuropein was tested against *L. major* promastigotes, and a median lethal concentration (LC_50_) of 50 µg/mL was obtained, indicating a cytotoxic effect and a reduction in parasite proliferation. The authors also researched the mechanism of action underlying the killing effect of oleuropein. The natural compound induced apoptotic death in these parasites in a time-dependent manner. Thus, oleuropein exhibits potent antiproliferative and leishmanicidal activity against *L. major* promastigotes (Elamin and Al-Maliki, 2014).

### 2.2 Sesquiterpene compounds trigger apoptosis, necrosis, autophagy, disruption of cell membranes, and exhibit immunomodulatory activity against *Leishmania* spp.

**Figure 3:**
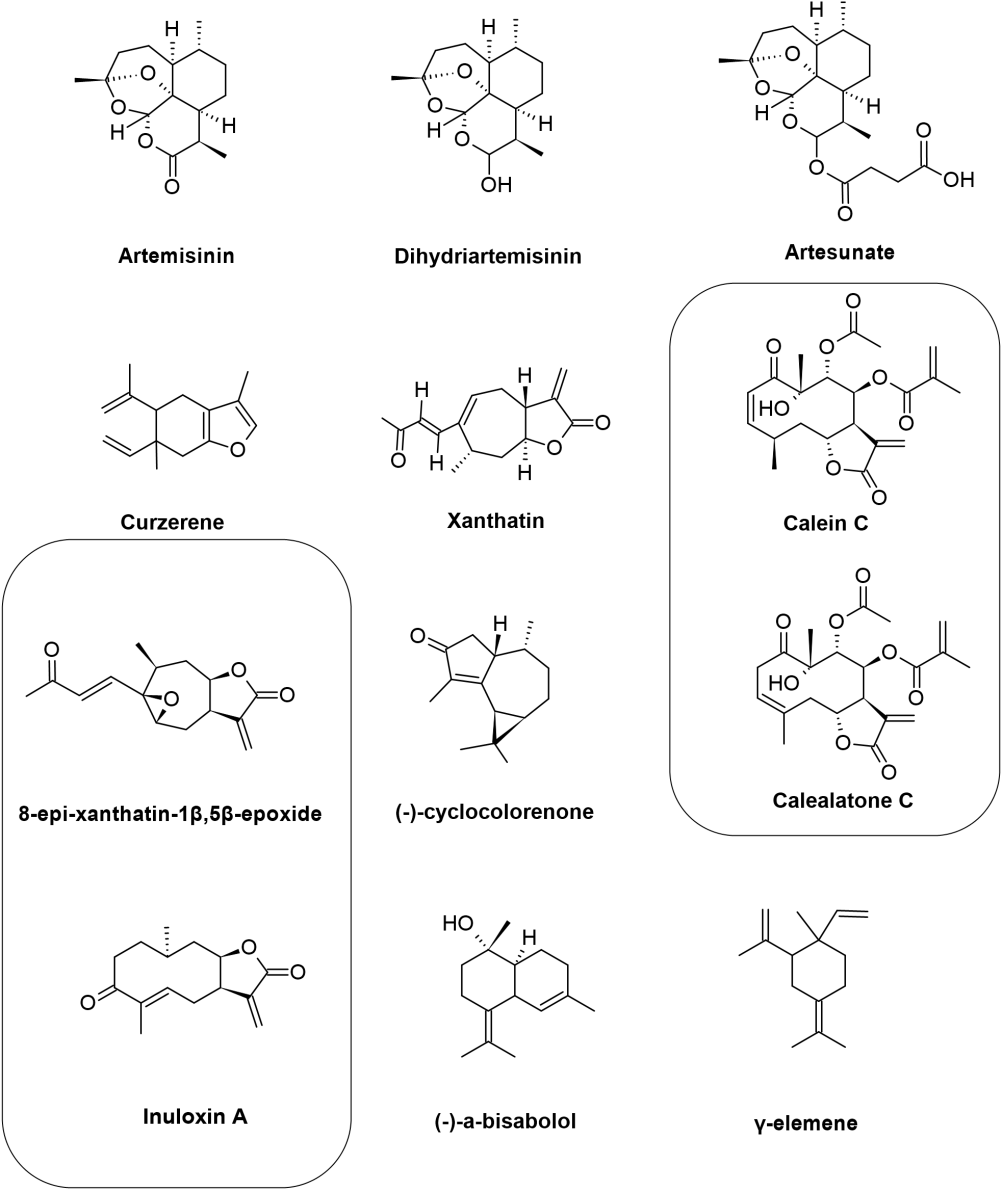
Chemical structures of the sesquiterpenes that showed activity against *Leishmania* spp. The figure shows the chemical structure of all sesquiterpenes present in the text that showed antileishmanial activity.

Sesquiterpenes (C_15_H_24_) are chemically characterized by having 15 carbon atoms, with assembly derived from three isoprenoid units, they are arranged in acyclic or mono, bi, tri or tetracyclic (**Figure 3**). Currently, a wide variety of complexes have been found that are metabolically derived from only 300 C15 hydrocarbon skeletons, however, the synthesis originates from a single substrate, farnesyl diphosphate (Chaturvedi, 2011; Fu et al., 2019; Moujir et al., 2020; Shu et al., 2021).

A sesquiterpene lactone used in therapy against malaria is artemisinin (ART), which is produced with leaves of the species *Artemisia annua* L. It is known that in *Plasmodium* spp. artemisinin increases ROS production by breaking endoperoxide binding, culminating in mitochondrial membrane potential depletion. According to the literature, ART has anti-*Leishmania* activity through a mechanism involving a decrease in mitochondrial membrane potential and ATP reduction, together with the formation of endoperoxide-mediated radicals, leading to secondary mitochondrial dysfunction, a similar process to what happens in *Plasmodium*. ART activity was evaluated in *L. donovani* promastigotes by De Sarkar et al. (2019), with an IC_50_ of 0.16 μM (De Sarkar et al., 2019). Similar, ART was assessed in *L. amazonensis* amastigotes by Machín et al. (2021), the IC_50_ was 15.0 μM and low index of cytotoxicity in macrophages in the highest concentration (200 μM). Based on this, in this study ART showed a high calculated selectivity index (> 13), which demonstrates safety for ART (Machín et al., 2021).

Dihydroartemisinin (DQHS) is a derivative of ART, with DQHS being an intermediate in the sodium borohydride reduction reaction. Like ART, it has antiplasmodial activity, and is believed to interact with the endoperoxide bond. Grazzia et al. (2021) demonstrated that *L. braziliensis* promastigotes and amastigotes showed greater sensitivity to DQHS when compared to ART. DQHS have an IC_50_ of 62.3 and 8.9 μM, respectively, and a selectivity index of 32%. The loss of viability of *L. braziliensis* was linked to the promotion of mitochondrial dysfunction and H_2_O_2_ production involved in oxidative stress. These series of events result in the inhibition of crucial parasite proteins, heme alkylation, loss of membrane integrity, and covalent binding to biological molecules (Grazzia et al., 2021).

Artesunate (ARS) is a more stable derivative with greater solubility from artemisinin. In a recent study, Intakhan et al. (2022) associated ARS e with AmB, with the aim of seeking treatments that minimize the toxicity of AmB. Promastigotes and amastigotes of *L. martiniquensis* were susceptible to ARS, being more selective for parasites when compared to the host (SI = 1.065) In promastigotes, the IC_50_ was 53,51 µM. With regard to ARS and AmB, five associations showed synergistic effects, ranging from mild to high synergism (combination index 0.28-0.92). Thus, it was possible to reduce the AmB dose by 9.7 times. This initial study is profoundly relevant as it encourages new less toxic formulations and increases the cure rate of patients in the clinic (Intakhan et al., 2022). Curzerene was originally isolated from the roots of *Curcuma longa*, however, curzerene is present in various essential oils from different species, including those belonging to the *Eugenia* genus. The molecular formula is described as C_15_H_20_O and molecular weight of 216.32. In the work by (Nunes et al., 2021b) curzerene was isolated from *Eugenia uniflora* L. and tested against *L. amazonensis*. In axenic amastigotes, effective concentration (EC_50_) was 2.56 µM and in promastigotes the IC_50_ was 3.09 µM. In addition, curzerene was not cytotoxic for RAW 264.7 cells, as it was more selective to the parasite in relation to the cell. Two main mechanisms of action were verified, direct and indirect. Indirectly, in intracellular amastigotes, curzerene activated macrophages, increasing lysosomal volume and nitric oxide (NO) levels. Directly in promastigotes, curzerene was able to induce cell death by apoptosis and necrosis simultaneously. In summary, the sesquiterpene curzerene holds great promise as a compound for leishmaniasis therapy (Nunes et al., 2021b).

Similar to these findings, Xanthatin, a sesquiterpene lactone isolated from the species *Xanthium strumarium* L. (Asteraceae), has described antimicrobial activity, as well as antiprotozoal activity, including anti-*Leishmania.* Against this, Akbari et al. (2021) examined the efficacy of Xanthatin on both amastigote and promastigote forms of *L. major*. The rate of infection and multiplication of amastigotes in J774 macrophages were 53% and 62% respectively, and IC_50_ was 1 μg/mL. Through metabolome analyses, it was discovered that Xanthatin is mainly involved in the alteration of sugar metabolism in general, with carbon metabolism being strongly affected, suggesting a decrease in parasite virulence (Akbari et al., 2021).

Calein and calaleactone C are sesquiterpene lactone extracted from the species *Calea pinnatifida* (Asteraceae). Bioactivity was tested by Caldas et al. (2019) in promastigotes, it exhibited an IC_50_ of 1.7 and 4.6 µg/mL for calein and calalelactone C, in the same order. In amastigotes, the EC_50_ was 31.73 and 27.18 µg/mL, respectively. By transmission electron microscopy, it was discovered that both sesquiterpenes acted on the mitochondria and nucleus, degrading both organelles of the parasite, in addition to promoting cell shrinkage and intracellular disorganization (Caldas et al., 2019).

Monteiro et al. (2022) identified the sesquiterpene (-) cyclocolorenone, through the fractionation of the ethanolic extract of leaves of *Duguetia lanceolata* (Annonaceae). The *in vitro* evaluation of the activity against promastigotes and amastigotes of *L. amazonensis* showed IC_50_ of 4.54 and 28.44 µM respectively and low cytotoxic activity against J774 macrophages, showing selectivity > 100 for promastigotes and > 32.2 for amastigotes. The authors attributed this effect to a tropism of the compound by the cell membrane of the parasite (Monteiro et al., 2022).

Bioguided fragments of *Inula viscosa* were used in *in vitro* assays against two strains of the genus *Leishmania*. During the process, several compounds with bioactive properties were identified, including two sesquiterpenes from the ethanolic extract. The bioactive compounds (F3) 8-epi-xanthatin-1β,5β-epoxide (IC_50_ 9.53 and 11.06 μM) and (F4) inuloxin A (IC_50_ 12.12 and 14.26 μM) demonstrated potential activity against the promastigote forms of *L. amazonensis* and *L. donatella*, compared to miltefosine (IC_50_ 6.48 and 3.31 μM, respectively) and a low toxicity in macrophage cell line (SI > 45), indicating a promising effect in combating the parasites. The leishmanicidal activity of the selected compounds against the amastigote stage of *L. amazonensis* was performed. The compound F3 exhibits a IC_50_ of 6.98 and the F4 of 0.64 μM (Zeouk et al., 2020).

Following the promising results of *in vitro* antiparasitic activities, the authors showed that after incubation for 24 h using IC_50_ of the compounds. In the case of *L. amazonensis* exposed to F3, a marked condensation of the chromatin structure was observed, which stood out compared to the control group. In addition, the penetration of propidium iodide through the cell membrane showed characteristics associated with cell death. However, in the case of *L. donovani*, when exposed to F3, it evidenced a process of apoptosis. The influence of the tested substances on mitochondria membrane potential (ΔΨm) was evaluated by means of the JC-1 fluorescence technique. When treatment was applied to *L. amazonensis* using compound 3, there was no breakdown in mitochondrial membrane integrity, however, compound 4 showed a remarkable depolarization. With regard to ATP production, a vital indicator of cellular activity, over the 24-hour period, treatment with compound 4 resulted in the most significant decrease in total ATP levels in *L. amazonensis*. The impact of this compound on *L. donovani* was more moderate. However, the inhibition of ATP synthesis in both parasites was less evident when treated with compound 3. Analysis of plasma membrane integrity was also performed, and both treatments with *Leishmania* spp. demonstrated a significant alteration in plasma membrane permeability. It was observed that treatment of *L. amazonensis* with compound 3 increased the generation of ROS, although this effect was not observed in *L. donovani* (Zeouk et al., 2020).

*Tunisian chamomile* EO has potent activity against *L. amazonensis* and *L. infantum*, the bioactive compound was elucidated by bioguided fractionation, isolation and identification of (-)-α-bisabolol which showed a low IC_50_. The effect of this sesquiterpene alcohol compound, which was separated, was analyzed with respect to its action on promastigote forms (16.0 and 9.5 μg/mL), *L. amazonensis* and *L. infantum*, respectively) Miltefosine showed higher activity against both parasites IC_50_ (5.3 and 4.3), respectively. Regarding amastigote forms, our results showed good activity of the sesquiterpene against *L. amazonensis and L. infantum* at lower IC_50_ (5.9 and 4.8 μg/mL, respectively) when compared to those for promastigote forms. The value of the 50% cytotoxicity concentration in macrophages was 31.9 μg/mL. The selectivity index (SI) was determined by using the IC_50_ values for intracellular amastigotes of both parasite species together with the CC_50_ value (SI = 5.5 and 6.7 for *L. amazonensis* and *L. infantum*, respectively). It was observed by them that the compound had the ability to trigger a mechanism of programmed cell death in the promastigote stage of the parasite. The apoptosis rates resulting from exposure to the sesquiterpene were 21.66% (IC_50_) and 40% (IC_90_) *for L. amazonensis*, while for *L. infantum* they were 17% (IC_50_) and 20% (IC_90_), after a 24-hour treatment period. The rates of cells that showed signs of death were modest (1.33% (IC_50_) and 2% (IC_90_) for *L. amazonensis*, and 9% (IC_50_) and 16% (IC_90_) for *L. infantum*), when compared to the dead cells in the positive control group (with rates of 16.66% and 61%). Additionally, the compound caused cell membrane damage, resulting in the external exposure of phosphatidylserine, as well as decreased total ATP levels and mitochondrial membrane potential (Hajaji et al., 2018).

In a comprehensive study involving isoflavonoids and terpenes, Araújo et al. (2022) succinctly showed the activity of the diterpene (-)-α-bisabolol against *L. infantum* and *L. amazonensis* promastigotes, with IC_50_ values 4,45 µg/mL and 4,77 µg/mL, respectively. In addition, the authors evaluated the mechanism of death of (-)-α-bisabolol in *L. infantum* and observed that the treatment was able to induce phosphatidylserine externalization, suggestive of apoptotic death (Araújo et al., 2022).

The study was carried out at the animal shelter of the Society for the Protection of Animals and Plants. Twelve dogs that already had natural infections were selected. These dogs were then randomly assigned to different groups to receive treatment. The therapeutic intervention consisted of administering (-)-α-bisabolol (six dogs) or meglumine antimoniate (six dogs). During the study, two of the dogs that had been treated with (-)-α-bisabolol were withdrawn from the project by their owners. It was possible to observe that (-)-α-bisabolol produced positive results in three of the four dogs, showing a gradual reduction in the parasite load, a phenomenon that became visible around the 60th day. At this point, there was an average decrease in the presence of parasites in the bone marrow (BM), lymph nodes (LN) and peripheral blood (PB). After the second series of treatment, which took place on day 120, there was an increase in the values related to the decrease in parasite load in the bone marrow (BM), lymph nodes (LN) and peripheral blood (PB). Dog 4, given the compound (-)-α-bisabolol, managed to completely eliminate the parasite by day 120. The treatment had the effect of increasing the production of gamma interferon (IFN-ɣ), which resulted in a combined response between the Th1 and Th2 pathways, with a trend towards Th1 cell-mediated immunity. On the other hand, variations in (IL-4) expression levels were less pronounced. When treatment involved the use of meglumine antimoniate, only one of the six dogs showed an effective response. Notably, in the dogs treated with meglumine antimoniate, IFN-ɣ expression levels were significantly lower. It is worth noting that treatment using the natural compound did not generate any manifestations of adverse symptoms, such as weight loss, diarrhea or vomiting. In addition, no significant changes were observed in the hematological tests or any signs of general toxicity. The average values of the biochemical parameters remained within the range considered normal (Corpas-López et al., 2018).

The essential oil was extracted from the leaves of the *Eugenia piauhiensis* plant, followed by analysis using gas chromatography coupled with mass spectrometry (GC-MS). In this study, it was possible to identify thirty-four different components present in the oil. Notably, the essential oil revealed a composition rich in sesquiterpene hydrocarbons. The main component identified was *γ-elemene*, which represented around 23.5% of the total composition, and its cytotoxicity and possible mechanisms of action were studied on promastigote and amastigote forms of *L. amazonensis*. Initially, the properties of *γ-elemene* were investigated in laboratory experiments that evaluated its effects on forms of the *L. amazonensis* parasite, including promastigotes (IC_50_ of 48.05 μM) and amastigotes (with IC_50_ of 39.44 μM). Additionally, the impact of the compound on J774.A1 cells was examined to determine its potential toxic effect (with IC_50_ of 1.043 μM). Notably, it was observed that *γ-elemene* had a lower toxicity towards macrophages compared to miltefosine, which is a drug widely used as a standard treatment. The SI (21.7) indicates the degree of preference of the substance for the parasite compared to mammalian cells, representing how many times the substance is more selective for the parasite. On the other hand, miltefosine showed the highest toxicity among the substances tested, with an SI value of 6.94 (Nunes et al., 2021a).

To assess the possible impacts of γ-elemene on the parasite membrane, membrane permeability tests were conducted. During these tests, a notable increase in the ability of fluorescent molecules to cross the membrane of promastigotes was noted when treated with *γ-elemene*. Once the efficacy and selectivity of *γ-elemene* against the *L. amazonensis* parasite had been confirmed, an *in vitro* infection model of J774A.1 macrophages was used to explore the interactions between the parasite and the host cell. By analyzing the effects of *γ-elemene* on reducing the infection rate in macrophages, an *in vitro* investigation was conducted to understand whether the anti-amastigote activity was linked to immunomodulatory effects. It was observed that treatment with *γ-elemene* resulted in an increase in the production of two immune mediators, TNF-α and IL-12. Notably, this action had no impact on the levels of the cytokines IL-6 and IL-10 under the conditions evaluated. Additionally, the production of de nitric oxide (NO) and ROS was examined in macrophages both in the presence and absence of promastigote forms of the *L. amazonensis* parasite. When the parasite was not present, there were no changes in NO production in macrophages treated with *γ-elemene*. However, when macrophages were stimulated with *L. amazonensis*, *γ-elemene* increased NO levels at a concentration of 25 μg/mL. Bacterial lipopolysaccharide (LPS) was used as a positive control and induced high NO production. When it came to ROS levels, treatment with γ-elemene at concentrations between 12.5 μg/mL and 25 μg/mL resulted in an increase in ROS levels only in *Leishmania*-stimulated macrophages. A compound called antimycin A, used as a positive control, also increased ROS levels both in the presence and absence of the parasite (Nunes et al., 2021a).

**Figure 4:**
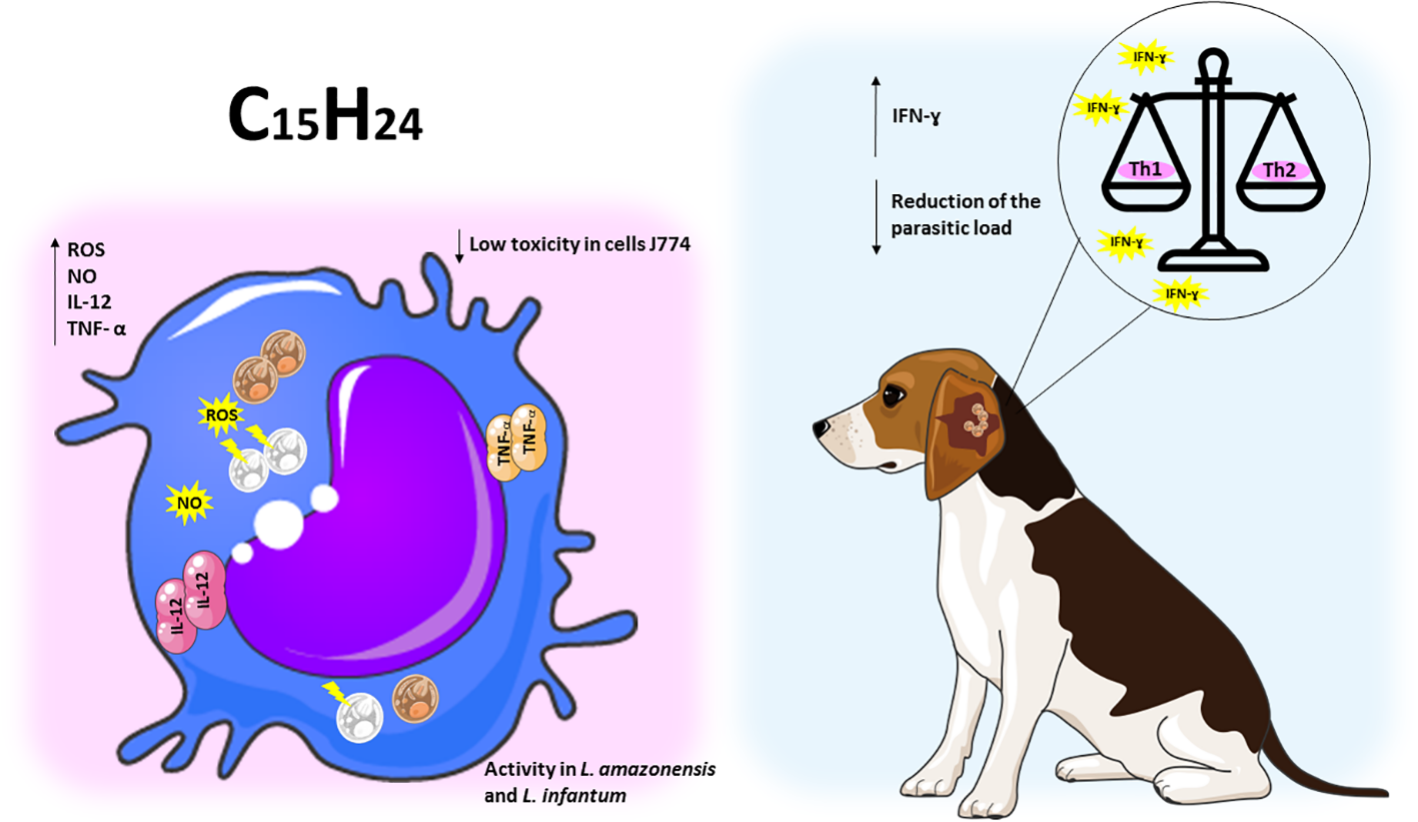
Sesquiterpene compounds show immunomodulatory activity against *Leishmania* spp. The representative scheme shows the activity of the sesquiterpene compounds against *L. amazonensis* and *L. infantum*. In the J774 cell line infected with *Leishmania* spp, the administration of sesquiterpene compounds promoted the increase of ROS, NO, IL-12 and TNF-a. In dogs infected with *L. infantum*, treatment with sesquiterpenes increased pro-inflammatory responses, leading to a balance of Th1/Th2 patterns and reduced parasite load.

Sesquiterpenes are compounds with promising properties in the treatment of leishmaniasis, a disease caused by parasites of the genus *Leishmania*. However, the efficacy of these compounds is affected by several mechanisms that lead to the death of amastigotes and promastigotes. Among the main death mechanisms of sesquiterpenes in leishmaniasis are the induction of apoptosis, necrosis, autophagy, and cell membrane disruption. These processes involve alterations in cell metabolism and structure, compromising parasite survival and proliferation (**Table 2** and **Figure 4**).

**Table 2:** Summary of the biological activity of sesquiterpenes.

Sesquiterpene	*Leishmania* spp	IC_50_ promastigotes	IC_50_ amastigotes	References
Artemisine	*L. donovani* (Visceral leishmaniasis) *L. amazonensis*	0.16 μM -	- 15.0 μM	(De Sarkar et al., 2019) (Machín et al., 2021)
Dihydroartemisinin	*L. braziliensis* (Cutaneous and mucosal leishmaniasis)	62.30 μM	8.90 μM	(Grazzia et al., 2021)
Artesunate	*L. martiniquensis* (Cutaneous and visceral leishmaniasis)	53.51 µM	–	(Intakhan et al., 2022)
Curzerene	*L. amazonensis* (Cutaneous leishmaniasis)	3.09 μM	2.56 μM	(Nunes et al., 2021b)
Xanthatin	*L. major* (Cutaneous leishmaniasis)	-	1 μg/mL	(Akbari et al., 2021)
Calein	*L. amazonensis* (Cutaneous leishmaniasis)	1.70μg/mL	31.73μg/mL	(Caldas et al., 2019)
Calaleactone	*L. amazonensis* (Cutaneous leishmaniasis)	4.60μg/mL	27.18μg/mL	(Caldas et al., 2019)
Cyclocolorenone	*L. amazonensis* (Cutaneous leishmaniasis)	4.54 μM	28.44 μM	(Monteiro et al., 2022)
8-epi-xanthatin- B, 5B epoxide	*L. amazonensis* (Cutaneous leishmaniasis) *L. donovani* (Visceral leishmaniasis)	9.53 μM 11.06 μM	6.98 μM -	(Zeouk et al., 2020)
Inuloxin A	*L. amazonensis* (Cutaneous leishmaniasis) *L. donovani* (Visceral leishmaniasis)	12.12 μM 14.26 μM	0.64 μM -	(Zeouk et al., 2020)
(-)-a-bisabolol	*L. amazonensis* (Cutaneous leishmaniasis) *L. infantum* (Visceral leishmaniasis)	16.00 μg/mL 4.77 μg/mL 9.50 μg/mL 4.45 μg/mL	5.90 μg/mL - 4.80 μg/mL -	(Hajaji et al., 2018) (Araújo et al., 2022)
y-elemene	*L. amazonensis* (Cutaneous leishmaniasis)	48.05 μM	39.44 μM	(Nunes et al., 2021a)

In column 1: type of sesquiterpene, column 2: species of Leishmania on which the compound showed activity, column 3: IC_50_ of promastigotes, column 4: IC_50_ of amastigotes and column 5: references.

### 2.3 Diterpenes induces structural changes, increased ROS and cytosolic calcium, triggering impairment of cholesterol synthesis and death in *Leishmania* spp.

Diterpenes (C_20_H_32_) consist of four isoprene units linked head-to-tail or tail-to-head, and have several possible structural subtypes. These terpenes are classified mainly on the basis of the number of rings present in their chemical structure. The major classes include acyclic (phytan), monocyclic (retinol - vitamin A), bicyclic (clerodane), tricyclic (abietane), tetracyclic (kaurene), as well as polycyclic and others structures (Eksi et al., 2020).

Grandiflorenic acid (ent-kaur-9(11),16-dien-19-oic acid) (GFA) is a major constituent of *Sphagneticola trilobata* (L.) Pruski (*Wedelia paludosa* DC.). In a study, this compound demonstrated leishmanicidal activity against promastigote forms of *L. amazonensis*, with an IC_50_ value of 25 nM. The mechanism of action involved the induction of ROS, exposure of phosphatidylserine, membrane permeabilization and mitochondrial depolarization. Treatment with GFA also resulted in a significant reduction in the percentage of infected macrophages at concentrations of 6.25 nM (88.33%) and 12.5 nM (93,3%). Furthermore, a decrease in the number of amastigotes per macrophage was observed at both concentrations, 6.25 nM (50.61%) and 12.5 nM (33.54%). The authors suggest that this reduction is associated with an increase in IL-10 levels and transferrin-bound total iron, which inhibits the parasite’s ability to acquire iron for its metabolism (Bortoleti et al., 2018).

Sugiol is a diterpenoid compound obtained from the bark of various trees belonging to the Cupressaceae family. In an experimental study, the efficacy of sugiol treatment was assessed at different time points: 24, 48 and 72 h, yielding IC_50_ values of 10,6 µg/mL, 5,5 µg/mL and 4,1 µg/mL, respectively. It was observed that the treatment with sugiol resulted in an elevation of cytosolic calcium levels, inducing autophagy in *L. infantum* promastigote forms within the initial 24 h. Concurrently, other cellular responses such as increased ROS, cellular shrinkage, and exposure of phosphatidylserine were also detected during the first 24 h of treatment. As time progressed, at 72 h, mitochondrial hyperpolarization was observed, attributed to the uptake of calcium by mitochondria, which further intensified ROS production and lipid peroxidation, ultimately leading to cell death through secondary necrosis in these parasites (Scariot et al., 2019).

Isolates obtained from the exudate of *Salvia uliginosa* were identified as diterpenoid α-hydroxy-β-isopropyl-benzo-quinones. Among these isolates, icetexane (ICT) and its derivative isocetexone (IsoICT) were evaluated for their activity against *L. amazonensi*s promastigotes. Both compounds demonstrated dose-dependent inhibition of the parasite, with IC_50_ values of 13 µM and 16 µM for IsoICT and ICT, respectively, after 48 h of treatment. Early on, at the 6 h time point, concentrations of 2 times the IC_50_ of IsoICT (26 µM) and ICT (32 µM) were found to increase the production of ROS, and by 24 h, DNA fragmentation was observed. Additionally, treatment with the IC_50_ of ICT at 48 h resulted in swelling, rounding, multiseptation of the cell body, and loss of body length and flagella in promastigotes forms. Importantly, both isolates showed no toxicity to RAW 264.7 macrophages and human erythrocytes, indicating their potential as selective agents against *L. amazonensis* (Cezarotto et al., 2019).

Diterpene acids are abundant constituents found in the resin of *Pinus* species, serving as a natural defense mechanism. In a study conducted by Gonçalves et al. (2018), a diterpene called dehydroabietic acid (DHA) obtained from *Pinus elliottii* resin was investigated. DHA showed an ability to decrease mitochondrial potential and increase ROS production in promastigote forms of *L. amazonensis*, leading to late apoptosis death, with an IC_50_ value of 40 µg/mL. In amastigote forms, treatment with DHA at concentrations of 40, 50 and 75 µg/mL led to ROS production, iron uptake, and downregulation of Nrf2 expression, indicating a pro-oxidant response at all tested concentrations (Gonçalves et al., 2018).

The diterpene 6,7-dehydroroyleanone (TrROY), derived from the essential oil of *Tetradenia riparia*, showed the ability to modulate cytokines upon infection of BALB/c peritoneal macrophages by *L. amazonensi*s. Treatment using several concentrations of TrROY (0.1, 1 and 100 µg/mL) decreased IL-4 production and promoted IL-12 increase. The authors chose the non-cytotoxic concentration (0.1 µg/mL) to perform the anti-amastigote assay and observed a 31% reduction in the infection rate of macrophages when treated (Terron-Monich et al., 2019).

Solidagenone (SOL), a labdan furan isolated from the plant *Solidago chilensis* Meyen (Asteraceae), showed the ability to inhibit *in vitro* promastigote forms of *L. amazonensis* at concentrations of IC_50_ (34.5 µM) and 2 x IC_50_ (69 µM). Treatment with SOL also caused imbalance in the parasite, leading to increased ROS production, mitochondrial depolarization, increased and cell volume, phosphatidylserine exposure, loss of plasma membrane integrity, and apoptosis-like death. Analyzing the intramacrophages forms, it was observed that SOL was able to reduce the percentage of infected THP-1 macrophages at all concentrations tested 20, 40, 80 and 160 µM, reducing by 52,61%, 71,03%, 81,53% and 90,90%, respectively. In addition, it reduced the number of amastigotes per macrophage by 34%, 43,40%, 51% and 67,30%, respectively. This parasite death occurred due to increased levels of IL-12p70, ROS and NO. In BALB/c mice infected with *L. amazonensis*, SOL showed potential effect in all concentrations tested (10, 50 and 100 mg/kg), reducing the parasite load. Regarding lesion size, in the final week of treatment, a decrease in lesion size was found at concentrations of 50 and 100 mg/kg, reducing 4,16 mm and 2,79 mm, respectively. It was also observed that SOL promoted a pro-oxidant/antioxidant balance controlling inflammation through immunomodulation and inducing wound healing and tissue repair, without causing gastric, hepatic and systemic toxicity in the animals (Bortoleti et al., 2021; Bortoleti et al., 2022).

Troncoso et al. (2023), studied 12-hydroxy-11,14-diceto-6,8,12-abietatrien-19,20-olide (HABTO) and 5-epi-icetoxone (ICTX), isolated from aerial parts of the *Salvia cuspidata* plant. Against promastigotes of *L. amazonensis*, they induced increased ROS levels and affected mitochondrial activity in all tested concentrations (4.3, 14.4 and 29.2 µM) in 48 h. In infected BALB/c mice, on the other hand, treatment with both using 1 mg/kg/day, decreased paw swelling, parasite load and splenic index. Furthermore, they induced a significant reduction in the levels of anti-*Leishmania* antibodies, IgG and IgG1, against *L. amazonensis* (Troncoso et al., 2023).

In another study, the biological activity of derivatives of ent-Beyerene, a diterpenoid found in species of the Asteraceae and Euphobiaceae families, which have been traditionally used in medicine for treating wounds and inflammation, was investigated. The ent-beyer-15-en-18-ol derivative isolated from the plant *Baccharis tola*, showed high toxicity to U-937 macrophages (a pro-monocity human myeloid leukemia cell line). However, it exhibited greater selectivity towards *L. braziliensis* amastigotes compared to host cells. Furthermore, treatment with creams containing different concentrations of beyerenol isomers 1 and 2 (I (1)-0.81%, II (1)-1.96% and III (2)-2.6%) in hamsters infected with *L. braziliensis* resulted in remarkable reduction in the size of skin lesions. Creams I and III showed comparable healing outcomes by the end of treatment, similar to the meglumine antimoniate control. Moreover, cream I induced a more significant inflammatory response, while cream III demonstrated a more pronounced reepithelialization process. Additionally, analysis of blood samples from the animals indicated no observed toxicity in any of the groups (Murillo et al., 2019).

Parasites belonging to the *Leishmania* genus exploit host membrane cholesterol to establish infection, leading to impaired antigen presentation and a compromised immune response against the parasite. In an investigation by Prakash et al. (2022), retinoic acid (RA) treatment in J774 macrophages infected with *L. donovani* restored cholesterol levels and reduced parasite burden. RA treatment upregulated the expression of Niemann-Pick C1 (npc1) and Niemann-Pick C2 (npc2) genes, involved in extracellular cholesterol uptake, while promoting immune response restoration by increasing inducible nitric oxide synthase (iNOS) expression and decreased arginase-1 levels. This suggests that combining RA with standard chemotherapy or as a preventive measure could be a significant advancement in the treatment and cure of VL patients (Prakash et al., 2022). Similarly, *L. donovani* infection impacts the expression of f sterol regulatory element-binding protein 2 (SREBP2), potentially resulting in reduced expression of 3-hydroxy-3-methylglutaryl-CoA (HMG-CoA) reductase (HMGCR), ATP-binding cassette transporter (ABCA1) and low-density lipoprotein receptor (LDLR). This indicates impairment of cholesterol synthesis, uptake and efflux in the infected cells. However, RA treatments reverses this phenomenon, leading to increased cellular cholesterol and reduced parasite burden (Prakash and Rai, 2022).

Vitamins, essential components in food, have gained attention in modern medicine for their efficacy against intracellular pathogens, including *Leishmania* sp. In a study by Gogulamudi et al. (2019), the combination of vitamin D3 with RA (Vit.D3/RA) (384 ng/30 ng/mice) and the isoprenoid chenodeoxycholic acid with RA (CDCA/RA) (78 ng/30 ng/mice) demonstrated a reduction in spleen, liver, and parasite load in BALB/c mice infected with *L. donovani*. Both treatment combinations activated a Th1 cellular response, with elevated levels of IL-1, IFN-γ and TNF-α, while inhibiting the Th2 response (IL-4 and IL-5). This suggests that Vit.D3/RA and CDCA/RA combinations have an immunomodulatory therapeutic role against leishmaniasis (Gogulamudi et al., 2019).

Another study evaluated the use of RA encapsulated in solid lipid nanoparticles (RA-SLN) to enhance intranasal (i.n.) vaccination with the complete *L. amazonensis* antigen (LaAg) in female BALB/c mice. Mice receiving the LaAg/RA-SLN vaccine demonstrated better outcomes in terms of reduced lesion growth and parasite burden compared to those receiving LaAg alone. Both vaccinated groups showed increased IL-12 production and decreased IL-4 and TGF-β production in the plantar pads, indicating a partially Th1-deviated immune profile. RA-SLN also induced elevated IL-10 levels and an increase in TregsCD4+Foxp3+ population in the mucosa, crucial for tolerance maintenance and inflammation prevention. These findings highlight the effectiveness and safety of RA-SLN as a tolerogenic adjuvant for leishmaniasis vaccination (Bezerra et al., 2019).

**Figure 5:**
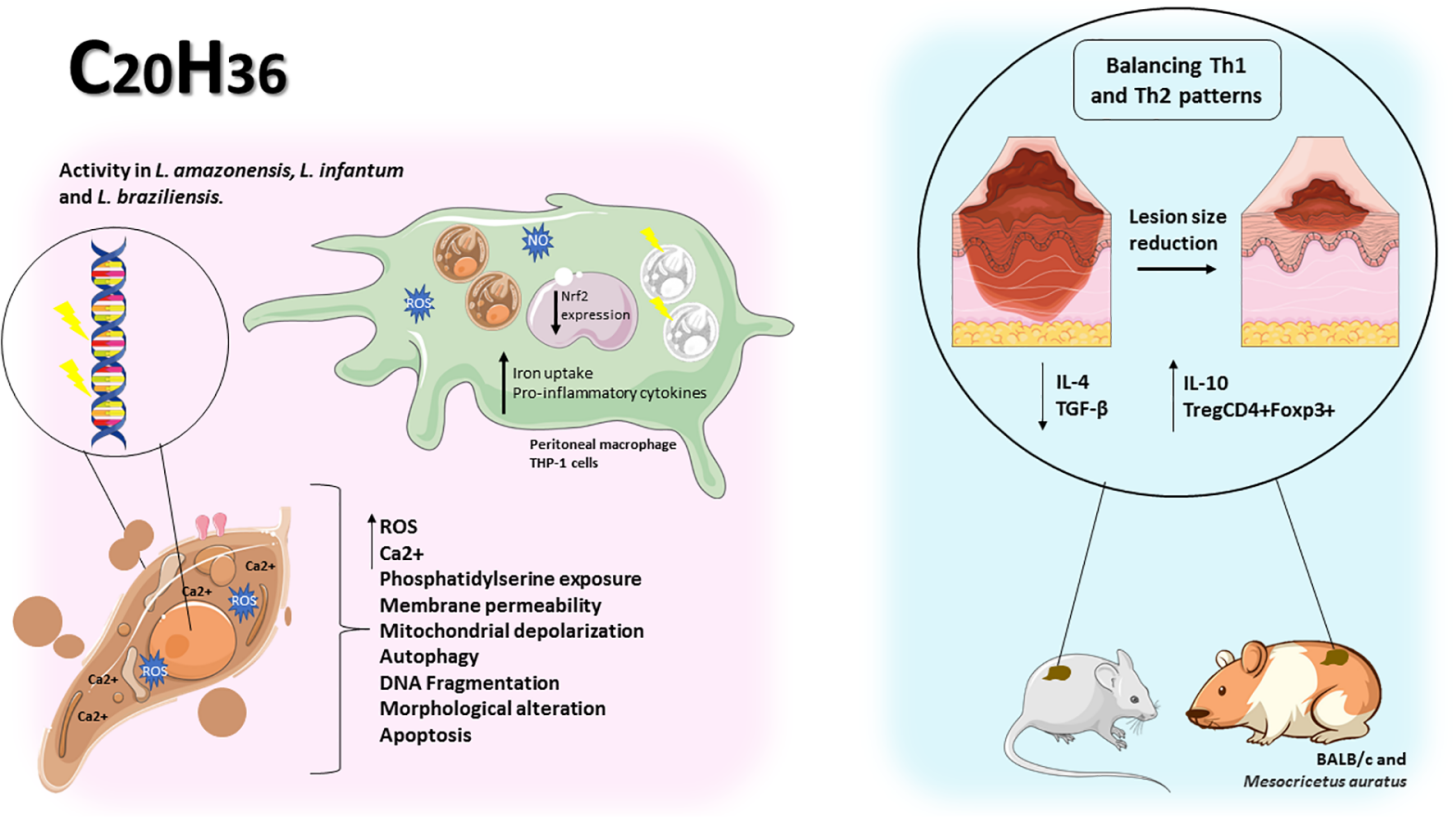
Diterpenes showed antileishmanial activity by causing change in parasite morphology, increased ROS and cytosolic calcium, plasma membrane permeability, mitochondrial depolarization, phosphatidylserine exposure, autophagy, and DNA fragmentation in promastigote forms. In peritoneal macrophages and THP-1 cells infected with Leishmania spp, iron uptake, pro-inflammatory cytokines and downregulation of Nrf2 were responsible for the reduction in the number of amastigotes per cell. In vivo diterpenes promote Th1/Th2 balance leading to reduction in lesion size and improved healing.

**Figure 6:**
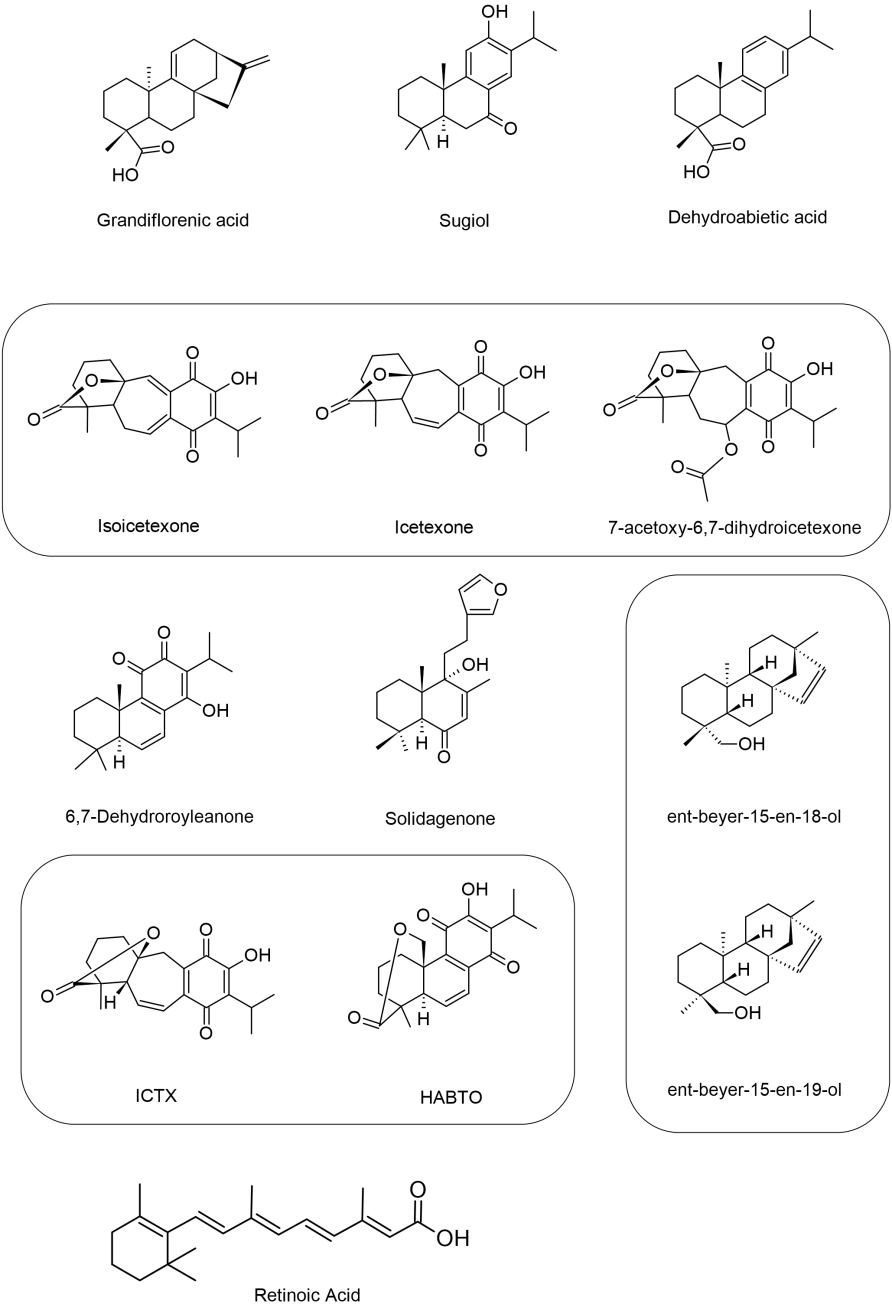
Structures of the diterpenes that showed activity against Leishmania spp. The figure shows the chemical structure of all diterpenes present in the text that showed antileishmanial activity.

In the study by Verma et al. (2019), blood samples were collected from patients with VL to measure serum levels of RA. The analysis revealed significantly reduced serum levels of this diterpene in the VL patients, suggesting that RA is an important dietary component. Additionally, inhibiting enzymes involved in RA synthesis (retinal dehydrogenase-1 (RALDH-1) and RALDH-2) in *L. donovani*-infected J774.1 macrophages increased arginase-1 and IL-10 levels while reducing iNOS, IFN-γ and TNF-α expression, resulting an anti-inflammatory (Th2) response detrimental to the host. However, inhibition of the RALDH-1 and RALDH-2 pathway actually exacerbates infection, indicating the crucial role of RA in the anti-*Leishmania* immune response and containment of the infection (Verma et al., 2019). The diterpenes show promising activities against *Leishmania*, a summary of the activities described above can be seen in **Table 3** and **Figure 5**. The chemical structure of the diterpenes mentioned in this section is shown in **Figure 6**.

**Table 3:** Summary of the biological activity of diterpenes.

Diterpene	*Leishmania* spp.	IC_50_ promastigotes	IC_50_ amastigotes	Reference
Grandiflorenic acid	*L. amazonensis* (Cutaneous leishmaniasis)	25 nM	0.96 nM	(Bortoleti et al., 2018)
Sugiol	*L. infantum* (Visceral leishmaniasis)	10.6 µg/mL (24h) 5.5 µg/mL (48h) 4.1 µg/mL (72h)	5.70 µg/mL	(Scariot et al., 2019)
Icetexane	*L. amazonensis* (Cutaneous leishmaniasis)	16 µM	–	(Cezarotto et al., 2019)
Isocetexone	*L. amazonensis* (Cutaneous leishmaniasis)	13 µM	–	(Cezarotto et al., 2019)
Dehydroabietic acid	*L. amazonensis* (Cutaneous leishmaniasis)	40 µg/mL	–	(Gonçalves et al., 2018)
6,7-dehydroroyleanone	*L. amazonensis* (Cutaneous leishmaniasis)	–	–	(Terron-Monich et al., 2019)
Solidagenone	*L. amazonensis* (Cutaneous leishmaniasis)	34.5 µM	9.5 µM	(Bortoleti et al., 2021)
12-hydroxy-11,14-diceto-6,8,12-abietatrien-19,20-olide	*L. amazonensis* (Cutaneous leishmaniasis)	–	–	(Troncoso et al., 2023)
5-epi-icetoxone	*L. amazonensis* (Cutaneous Leishmaniasis)	–	–	(Troncoso et al., 2023)
Ent-beyer-15-en-18-ol	*L. braziliensis* (Cutaneous and mucosal leishmaniasis)	–	–	(Murillo et al., 2019)
Retinoic acid	*L. donovani* (Visceral leishmaniasis)	–	–	(Prakash et al., 2022) (Prakash and Rai, 2022)
Retinoic acid encapsulated	*L. amazonensis* (Cutaneous leishmaniasis)	–	–	(Bezerra et al., 2019)

In column 1: type of diterpene, column 2: species of Leishmania on which the compound showed activity, column 3: IC_50_ of promastigotes, column 4: IC_50_ of amastigotes and column 5: references.

### 2.4 Triterpenes exert immunomodulatory activity, reduce the Hsp90 protein and the parasite load in *Leishmania* spp.

**Figure 7:**
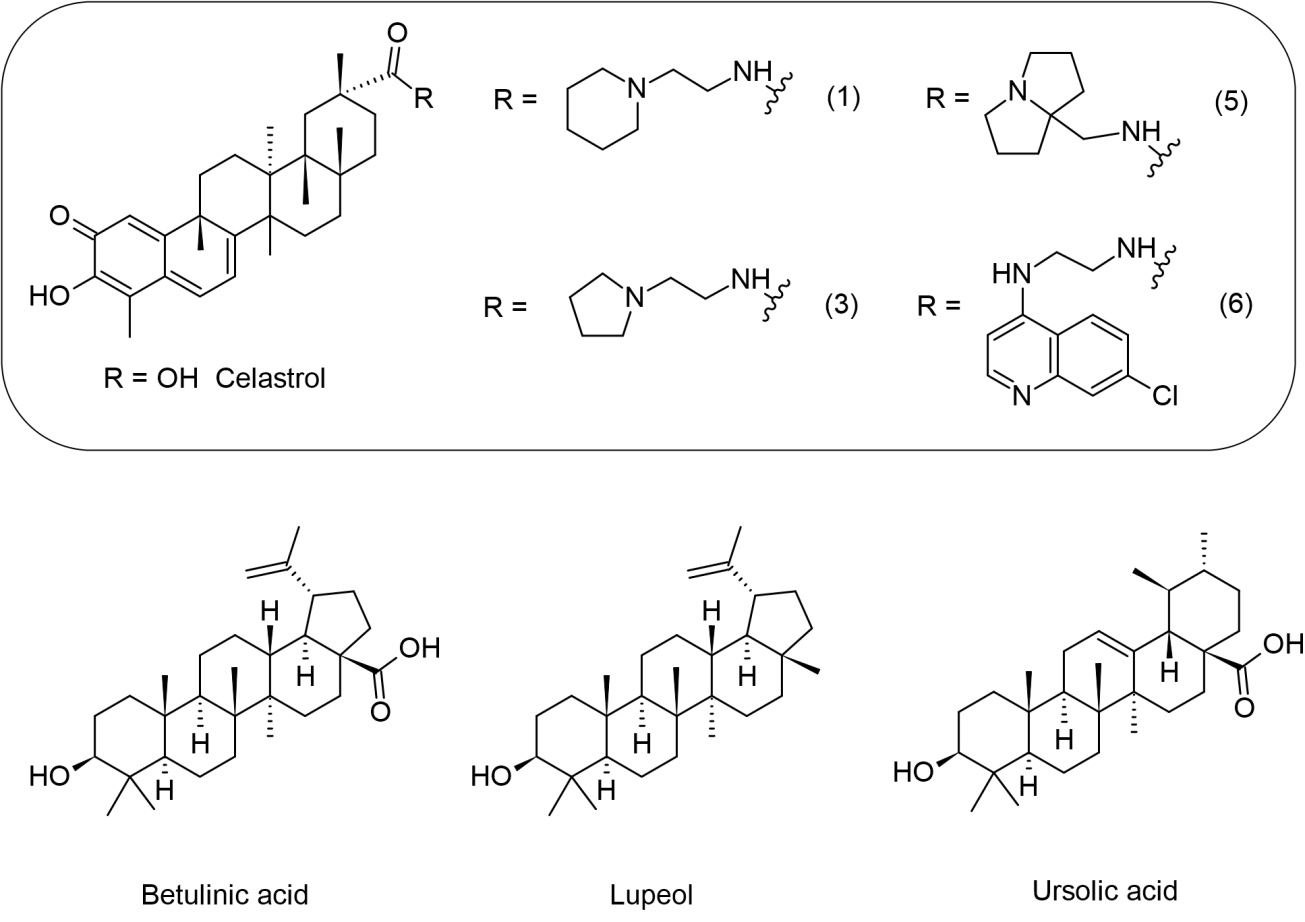
Chemical structures of the triterpenes that showed activity against *Leishmania* spp.

By definition, triterpenes (C_30_H_48_) are polycyclic compounds, representing one of the largest classes of natural products with over 4000 different compounds identified (Silva et al., 2020). Based on their chemical structure, they can be divided into acyclic (squalene group), monocyclic (achilleol A), bicyclic (pouoside A), tricyclic (lansioside A), tetracyclic (lanostanes, euphanes, tirucallanes, cucurbitanes, dammaranes and baccharanes), and pentacyclic triterpenoids (cycloartanes, ursanes, oleananes, lupanes, friedelanes, hopanes, and serratanes), these cyclic triterpenes can be further converted into various metabolites, including steroids, saponins, limonoids, and other chemically related compounds (**Figure 7**) (Bachořík and Urban, 2021; Miranda et al., 2022).

Morphological differentiation of *Leishmania* parasite processes is associated with heat shock proteins, including a specific human variant of the ubiquitous protein chaperone 90 kDa Heat Shock Protein (Hsp90) (Hombach-Barrigah et al., 2019). Celastrol (CE), a natural quinone methide triterpene from *Tripterygium wilfordii*, is thought to interact with Hsp90. A screening of eleven new basic celastrol carboxamides synthetized using celastrol as a lead compound revealed that CE carboxamides 1, 3, 5, and 6 had the most significant growth inhibitory activity against two different species of *Leishmania* promastigotes (*L. infantum and L. tropica*) and a reduced profile of a specific cytotoxicity for human microvascular endothelial cell line (HMEC-1), THP-1 and the bone marrow-derived murine line (BMDM). These CE carboxamides (compounds 1, 3, 5, and 6) also showed leishmanicidal activity on intramacrophage amastigotes of *L. infantum*. Therefore, regulation of the Hsp90 chaperone normally depends on ATP hydrolysis to allow heat-shock or pro-survival responses. In the same study, compound 3 was found to be the most active compound with the ability to inhibit the ATPase activity from *L. braziliensis* protein chaperone Hsp90 through a non-ATP-competitive mode of action. These triterpenes derivates may be a promising target for Hsp90 in the leishmanicidal activity (Bassanini et al., 2021).

Antileishmanial efficacy against *L. donovani* may also benefit from treatment with betulinic acid (BA), a pentacyclic triterpenoid usually isolated from *Betula alba* bark. Halder et al. (2018) investigated the effect of BA, loaded on uniformly spherical PLGA nanoparticles (BANPs; diameter 187.5 nm) coated with lactoferrin (Lf-BANPs), in BALB/c-derived peritoneal macrophages uninfected or infected with *L. donovani*. The reduction in the number of amastigotes per 100 macrophages was more significantly improved by treatment with Lf-BANPs (2.5 µg/mL) than by treatment with BA (2.5 µg/ml) and BANP (2.5 µg/mL) alone. Moreover, compared with BA and BANP, the combined treatment of BA and lactoferrin (Lf-BANPs) resulted in a significant induction of nitrite production in macrophages infected with *L. donovani* and an increase in the levels of iNOS and IL-12, while the levels of a pro-parasitic cytokine IL-10 decreased. The authors concluded that Lf-BANPs have an immunomodulatory effect and thus could be a potential candidate for the control of leishmaniasis (Halder et al., 2018).

The synergistic effect between suboptimal doses of lupeol (3 mg/kg body weight/day), a triterpenoid from *Sterculia villosa*, and AmB (0.1 mg/kg body weight/day) in *L. donovani*-infected BALB/c mice was investigated by Das et al. (2021). The lupeol-AmpB combination was able to significantly reduce the parasitic burden in both the liver and spleen of the infected mice, in contrast to treatment with lupeol or AmpB alone. The results also showed that the lupeol-AmpB combination induced the expression of iNOS2 along with enhanced formation of NO from splenocytes in *L. donovani*-infected mice. Further analysis showed that lupeol-AmpB treatment can modulate the activation of p38 mitogen-activated protein kinase (p38MAPK) and promote a decrease in phosphorylation of extracellular signal-related kinase (ERK -1/2), leading to an increase in IFN-γ and IL-12, while reducing the production of IL-10 and TGF-β. These results suggest that lupeol has an immunomodulatory and anti-leishmanial activity that elicits a Th1 cytokine response, downregulates immunosuppressive cytokines, and restores host immunity. This association with AmB potentiates the effect in the treatment of visceral leishmaniasis (VL) (Das et al., 2021).

In a similar approach, a comparative analysis of the triterpenoid lupeol and AmB revealed differences in immunomodulatory responses against *L. donovani*. Interestingly, lupeol (CC_50_ 960.04 μg/mL) was negligible and less cytotoxic to THP-1 cells than AmB (IC_50_ 27.73μg/mL) and was responsible for anti-promastigote activity by inducing cell cycle arrest in the sub-G0/G1 phase. The parasite load in the spleens of BALB/c mice AmB-treated animals decreased (98.16%), but the efficacy of treating infected animals with lupeol at two concentrations (25 and 50 mg/kg body weight) was significantly higher. In addition, infected animals treated with lupeol showed delayed hypersensitivity response (DTH), induction of iNOS-mediated NO and ROS production, expression of NF-κB, activation of CD4+ T and CD8+ T cells, subsequently an increase in Th1 (IL-12, TNF-α, IFN-γ, IL-17), whereas Th2 (IL-4, IL-10) cytokines decreased. Thus, lupeol was found to be a potential agent against VL (Kaur et al., 2019).

Corroborating with these studies, De Jesus et al. (2021) examined the effects of the triterpenes betulin (Be), lupeol (Lu), and ursolic acid (UA) in golden hamsters (*Mesocricetus auratus*) infected with *L. infantum*. In this study, all triterpenes were active in inhibiting the promastigote form of *L. infantum* growth, but only Lu and UA were more effective at impairing intracellular amastigote forms compared to Be and miltefosine. The triterpene treatment on infected macrophages did not produce quantifiable levels of NO, although Lu increased the levels of hydrogen peroxide in a dose-dependent manner. Analyses showed that the triterpenes induced morphological changes, in which Lu and UA decrease ΔΨm in a time-dependent manner. When administered to infected hamsters, UA or Lu (2.5 mg/kg) significantly reduced the parasitism in the spleen and liver, increased IFN-γ levels, and only lupeol exhibited iNOS gene expression when compared to the infected control. Taken together, the authors suggest that the leishmanicidal activity of triterpenes is mediated by immunomodulation (De Jesus et al., 2021).

The multifunctional capabilities of triterpenoids are widely recognized, and the efficacy of ursolic acid (UA) against promastigotes and intracellular amastigotes of *L. amazonensis* and *L. infantum* in acute and chronic models has been evaluated. In this study, UA was found to have more significant activity in amastigotes than in promastigotes compared with the reference drug used, miltefosine. To assess the effect in the acute infection model of VL, infected male BALB/c mice were treated intraperitoneally with UA at a dose of 5 mg/kg daily for seven consecutive days. The growth of spleen and liver parasites was reduced by 99.83% and 99.78%, respectively. This result was also observed in the chronic infection model of VL, in which male golden hamsters (*Mesocricetus auratus*) showed a 58% and 79% reduction in the number of parasites in the spleen and liver, respectively. In addition, topical administration of UA ointment (0.2% and 0.5%) and cream (0.5%) used for 28 days against *L. amazonensis* in a chronic infection model of cutaneous leishmaniasis (CL) reduce lesion size. Immunological analyses were performed indicating the ability of UA to modulate cytokine production. The results of this study indicate that UA may lead to promising treatments for cutaneous and VL (Bilbao-Ramos et al., 2020).

Regarding the leishmanicidal activity of the triterpene ursolic acid (UA), results from another study show that UA-loaded nanostructured lipid carriers (UA-NLC) increase efficacy of this triterpene in experimental VL caused by *L. infantum*. Healthy golden hamsters (*M. auratus*) treated with UA, NCL, and UA-NLC showed no morphological changes in visceral organs (spleen, lungs, and heart), and the levels of AST (aspartate aminotransferase), ALT (alanine aminotransferase), urea, and creatinine were normal. However, low numbers of amastigote forms were observed in the spleen and liver of infected hamsters treated with 1.25 or 5.0 mg/kg UA-NLC or UA. In the analysis of immune responses, the groups treated with UA-NLC (1.25 or 5.0 mg/kg) had higher IFN-γ levels and higher expression of the iNOS gene than animals treated with UA at the same doses, and only hamsters treated with 5.0 mg/kg UA-NLC produced a significant amount of antileishmanial IgG and IgG2. Given the low solubility of this triterpene, which limits its efficacy, the use of a UA-loaded nanocarrier allows for a more significant response for the treatment of VL (Jesus et al., 2021).

**Figure 8:**
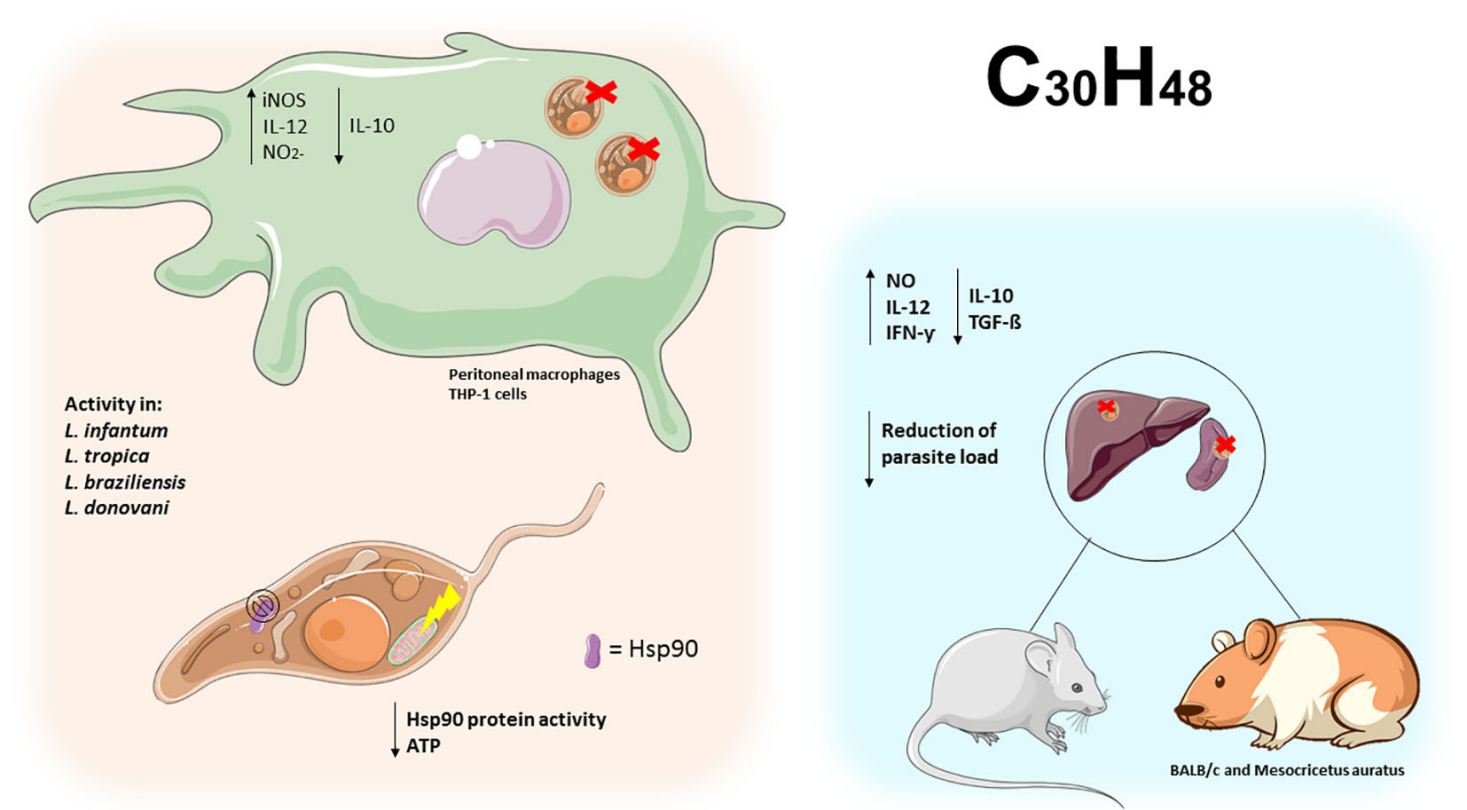
Triterpenes exert activity on the *Leishmania* Hsp90 protein leading to ATP depletion in promastigotes. In peritoneal macrophages and infected THP-1 cells, triterpene compounds increased IL-12 and NO_2_- and decreased IL-10, leading to elimination of intracellular amastigotes. In BALB/C mice and hamsters, triterpene compounds increased Th1 cytokines and decreased Th2 cytokines, leading to parasite clearance in the spleen and liver of infected animals.

Collectively, these studies demonstrate the promising potential of triterpenes in immunomodulating the immune response and inducing parasite death (**Table 4** and **Figure 8**). However, it is important to note that most of these findings are derived from *in vitro* experiments. Therefore, further investigations are necessary to comprehensively comprehend the potential mechanisms of action of terpene compounds and their future clinical applications.

**Table 4:** Summary of the biological activity of triterpenes.

Triterpenes	*Leishmania* spp.	IC_50_ promastigotes	IC_50_ amastigotes	References
Celastrol	*L. infantum* (Visceral leishmaniasis) *L. tropica* (Cutaneous leishmaniasis) *L. braziliensis* (Cutaneous and mucosal leishmaniasis)	0.06 µM (1) 0.06 µM (3) 0.12 µM (5) 0.09 µM (6) 0.09 µM (1) 0.08 µM (3) 0.12 µM (5) 0.14 µM (6) - - - -	0.19 µM (1) 0.13 µM (3) 0.52 µM (5) 0.66 µM (6) - - - - - - - -	(Bassanini et al., 2021)
Betulinic acid	*L. donovani* (Visceral leishmaniasis)	–	–	(Halder et al., 2018)
Lupeol	*L. donovani* (Visceral leishmaniasis) *L. infantum* (Visceral leishmaniasis)	25.49 µg/mL (72 h) 4 µM	25.49 µg/mL^-1^ 17.50 µM	(Kaur et al., 2019) (De Jesus et al., 2021)
Betulin	*L. infantum* (Visceral leishmaniasis)	133 µM	–	(De Jesus et al., 2021)
Ursolic acid	*L. infantum* (Visceral leishmaniasis) *L. amazonensis* (Cutaneous leishmaniasis)	8 µM 20.9 µM 17 µM	3 µM 6.70 µM 2.24 µM	(Bilbao-Ramos et al., 2020)

In column 1: type of triterpene, column 2: species of Leishmania on which the compound showed activity, column 3: IC_50_ of promastigotes, column 4: IC_50_ of amastigotes and column 5: references.

This work aimed to show the most recent studies involving the use of terpene compounds against *Leishmania* species. In view of the challenges currently faced in the treatment of leishmaniasis, it is important that experimental studies with natural compounds are encouraged, so that better effects are found and patients suffer fewer and fewer adverse effects. Unfortunately, most of the studies presented only show the compounds’ activity *in vitro*, but we believe that a review could help to promote interest *in vivo* and clinical studies with the terpenes mentioned in this text.

The purpose of this work was to present recent research on the use of terpenic compounds against different species of *Leishmania*. Considering the current obstacles in the treatment of leishmaniasis, it is of utmost importance to encourage experimental research with natural compounds. This approach aims to achieve more effective effects while minimizing adverse effects on patients. Most of the studies presented here are limited to demonstrating the *in vitro* activity of the compounds. However, we believe that an analysis of these effects could stimulate interest in clinical and *in vivo* research related to the terpenes mentioned in this article.

## 3 Conclusion

The exploration of natural bioactive compounds for antileishmanial drug development is an expanding field of research with an increasing number of species, extracts and new molecules being investigated. The interesting compounds reviewed here highlight a rich source of new molecules against different *Leishmania* spp. In this study, terpenes including mono-, sesqui-, di- and triterpenes were found to exert antiproliferative and parasite-killing activities mainly through the generation of ROS, NO, exposure of phosphatidylserine, mitochondrial and DNA damage, autophagy, iron uptake and production of pro-inflammatory cytokines. In addition, according to the data reviewed, the triterpene class has the largest number of molecules studied and high leishmanicidal activity, mainly by promoting an immunomodulatory effect, with an increased Th1 cytokine profile and reduced Th2. In this scenario, it is possible that these compounds can be used as a source of new treatments for leishmaniasis in the future.

## Author contributions

AR: Conceptualization, Investigation, Methodology, Project administration, Supervision, Writing – original draft, Writing – review & editing. AC: Writing – original draft, Writing – review & editing. MG: Conceptualization, Formal Analysis, Methodology, Writing – original draft, Writing – review & editing. VC: Investigation, Writing – original draft, Writing – review & editing. MD: Writing – original draft, Writing – review & editing. YS: Writing – original draft, Writing – review & editing. EC: Writing – original draft, Writing – review & editing. MM: Writing – original draft, Writing – review & editing. AN: Writing – original draft, Writing – review & editing. MP: Writing – original draft. NS: Writing – original draft. RM: Writing – original draft. DB: Project administration, Supervision, Writing – review & editing. WP: Supervision, Writing – review & editing. FB: Funding acquisition, Writing – original draft, Writing – review & editing.
